# Single-center task analysis and user-centered assessment of physical space impacts on emergency Cesarean delivery

**DOI:** 10.1371/journal.pone.0252888

**Published:** 2021-06-10

**Authors:** Kenji T. Sotto, Laura C. Hedli, Lillian Sie, Kimber Padua, Nicole Yamada, Henry Lee, Louis Halamek, Kay Daniels, Dan Nathan-Roberts, Naola S. Austin

**Affiliations:** 1 San José State University, San Jose, California, United States of America; 2 The Safety Learning Laboratory for Neonatal and Maternal Care, Stanford University, Stanford, California, United States of America; 3 Department of Pediatrics, Stanford University, Stanford, California, United States of America; 4 Department of Obstetrics and Gynecology, Stanford University, Stanford, California, United States of America; 5 Department of Anesthesiology, Perioperative and Pain Medicine, Stanford University, Stanford, California, United States of America; Lausanne University Hospital: Centre Hospitalier Universitaire Vaudois (CH), SWITZERLAND

## Abstract

Cesarean delivery is the most common surgery performed in the United States, accounting for approximately 32% of all births. Emergency Cesarean deliveries are performed in the event of critical maternal or fetal distress and require effective collaboration and coordination of care by a multidisciplinary team with a high level of technical expertise. It is not well understood how the physical environment of the operating room (OR) impacts performance and how specialties work together in the space.

**Objective:**

This study aimed to begin to address this gap using validated techniques in human factors to perform a participatory user-centered analysis of physical space during emergency Cesarean.

**Methods:**

This study employed a mixed-methods design. Focus group interviews and surveys were administered to a convenience sample (n = 34) of multidisciplinary obstetric teams. Data collected from focus group interviews were used to perform a task and equipment analysis. Survey data were coded and mapped by specialty to identify reported areas of congestion and time spent, and to identify themes related to physical space of the OR and labor and delivery unit.

**Results:**

Task analysis revealed complex interdependencies between specialties. Thirty task groupings requiring over 20 pieces of equipment were identified. Perceived areas of congestion and areas of time spent in the OR varied by clinical specialty. The following categories emerged as main challenges encountered during an emergency Cesarean: 1) size of physical space and equipment, 2) layout and orientation, and 3) patient transport.

**Conclusion:**

User insights on physical space and workflow processes during emergency Cesarean section at the institution studied revealed challenges related to getting the patients into the OR expediently and having space to perform tasks without crowding or staff injury. By utilizing human factors techniques, other institutions may build upon our findings to improve safety during emergency situations on labor and delivery.

## Introduction

Cesarean delivery is the most common surgery performed in the United States, accounting for approximately 32% of all births [1, 2]. The standard for urgent Cesarean is delivery of the fetus within 30 minutes after the obstetrician decides that operative delivery is indicated [3]. This recognizes that time is required to mobilize the obstetric, anesthesia and pediatric teams, move the patient to an operating room (OR), ensure an appropriate level of anesthesia, and deliver the fetus; however, this timing is challenging to achieve [4–6].

Emergency Cesarean deliveries are performed when an unforeseen complication arises during the labor process and time is of the essence. This complex and stressful procedure requires effective communication, coordination of multidisciplinary teams, technical expertise, and the ability to make decisions quickly and perform tasks efficiently under time pressure. Emergency Cesarean deliveries require a high level of human and system performance, and it is not well understood how the design of the physical environment assists or impedes performance.

Previous research has found significant variation among existing hospitals in labor and delivery OR size and design layout despite similarities in equipment and processes [7, 8]. This work suggests emergency Cesarean deliveries are performed in spaces designed based on available guidelines [9–11], building codes [12], and/or local constraints, but the spaces have not been fully optimized or standardized equivalently to spaces in other high-reliability organizations (e.g., cockpits in aviation, control rooms in nuclear power plants, etc.) [13]. OR design has been reviewed [14], but Cesarean OR design has not been specifically studied.

Individual responsibilities of various healthcare professionals (i.e., obstetricians, anesthesiologists, pediatricians/neonatologists, obstetric and pediatric/neonatal nurses, OR technicians, etc.) during emergency Cesarean delivery are well described in clinical practice guidelines [15–19]. Interdependence between specialties is frequently mentioned; however, the individual and collective space utilization and equipment usage of healthcare professionals during emergency Cesarean is not well understood in the current literature [20–31].

This study aimed to begin to address this gap using validated techniques in human factors to perform a participatory user-centered analysis of physical space during emergency Cesarean.

This approach is intended to inform future research on obstetric OR design choices and advance the goal to develop an ideal physical layout for emergency Cesarean deliveries.

## Materials and methods

This study employed a mixed-methods design exploring clinician perspectives at one institution regarding impacts of physical design on quality of care and safety during emergency Cesarean delivery. This study was approved by the Institutional Review Board (IRB) of Stanford University.

### Human subjects

A convenience sample of 34 clinicians from multidisciplinary obstetric teams at Lucile Packard Children’s Hospital Stanford (LPCH) were directly recruited and included in this study following the conclusion of an ongoing simulation-based obstetric crisis resource management program. Subjects included a diverse group of anesthesiologists, obstetricians/gynecologists, pediatricians/neonatologists, obstetric (OB) nurses, and OB surgical technicians.

### Tool development

The semi-structured interview and survey instruments were developed using an iterative participatory design approach. Potential items for the survey and semi-structured interview instruments were identified by three authors (KS, DN-R, and NA) from a tour of the three ORs dedicated for obstetric surgical procedures (OR-A, OR-B, and OR-C) at LPCH, informal interviews of clinicians from the multidisciplinary labor and delivery team, observations of three elective Cesareans lasting approximately 90 minutes each, and a literature review. Both the survey and interview instruments were iteratively refined following pilot testing with several clinicians including an anesthesiologist, obstetrician, and OR circulating nurse. The final survey (S1 Appendix) consisted of 18 items including multiple choice, short answer, and Likert-scale questions. The survey assessed subjects’ perspectives on OR preference, whether OR orientation, OR size, and availability of medical equipment would facilitate the speed of an emergency Cesarean delivery, and issues encountered during emergency Cesarean delivery. Preliminary questions asked participants about their demographic, specialty, and experience. A graphic depicting the layout of OR-B was also included for subjects to indicate where most of their time was spent (Item 6 in S1 Appendix) and areas of congestion (Item 7 in S1 Appendix). OR-B was selected based on being the median amongst the three ORs in terms of size, layout, and location within the unit.

The semi-structured interview (S2 Appendix) consisted of 11 items. Prompts assessed problems encountered during an emergency Cesarean delivery, problems with patient transport from labor and delivery rooms to the OR, the impact of OR size on workflow, and suggested solutions. Several items assessed tasks performed and equipment used to perform those tasks during an emergency Cesarean delivery.

### Data collection protocol: Focus groups

Seven, sixty-minute focus groups were conducted between May 2018 and March 2019 at LPCH. Five of these focus groups were scheduled directly after a multidisciplinary simulation-based obstetric crisis resource management program for nurses, anesthesiologists, and obstetricians. Two focus groups were conducted with pediatricians and OR technicians separately. All subjects were asked for verbal consent to participate in the survey and focus group interviews. Not all subjects consented to audio recording. Each subject completed an IRB Research Information Sheet (S3 Appendix), a Provider Demographic Questionnaire (S4 Appendix), and survey (S1 Appendix). The focus group interview, using the semi-structured interview instrument (S2 Appendix), was facilitated by one of two authors (KS and DN-R). Each subject was asked to self-identify their specialty and training status (Item 1 in S2 Appendix) in order to facilitate coding responses. At least one notetaker was present to document subject commentary. The task analysis and equipment timeline from the pilot test was iteratively developed with each focus group. Data collection ceased when saturation was reached.

### Data analysis

#### Characteristics of labor and delivery units

The floorplan of the labor and delivery unit, accurate measurements of the floorplan of each OR, and measurements, layout, and orientation of equipment within each OR were captured during the tour.

#### Task analysis

Task analysis provides a framework for breaking down a complex multidisciplinary situation into concise pieces that can be individually evaluated for safety and efficiency. Tasks and equipment identified by the focus groups were grouped by specialty. The number of tasks to be performed by each specialty were counted. Tasks that required individuals from multiple specialties and stopping points were identified.

#### Perceived areas of congestion and time spent by specialty

Survey items 6–7 (S1 Appendix) were coded and mapped by specialty in order to identify areas where subjects perceived most time is spent and self-reported areas of congestion, respectively.

#### Overlay of perceived areas of congestion and task location

A compiled map was developed in order to determine areas of congestion that were reported by multiple specialties. The tasks identified from the task analysis were plotted on top of the compiled map to determine if there was a correlation between the two.

#### Qualitative analysis

Open ended questions assessing challenges encountered during an emergency Cesarean delivery and proposed solutions from both surveys and interviews were analyzed using a qualitative analytic approach [32]. A complete thematic analysis was not chosen because audio recordings and transcripts were not captured in all of the sessions. Instead an adapted version of methodology by Braun and Clarke [32] was performed by one author (KS). Exploratory themes were developed inductively through line-by-line coding of each subject’s responses using *QSR International NVivo* software. Similar themes were then grouped into descriptive categories and adjudicated by the team as necessary.

#### Quantitative analysis

Exploratory quantitative data from the surveys were analyzed using *IBM SPSS* statistical software, version 25. Kruskal-Wallis H tests were used to analyze whether there were statistically significant differences in rating of 1) orientation and 2) size of each of the three ORs, and 3) availability of equipment between specialties. Given the small sample size from one institution, quantitative analyses are not included as primary findings and included as (S1 and S2 Tables).

## Results

### Characteristics of study subjects

A convenience sample of 34 clinicians from multidisciplinary obstetric teams at LPCH participated in seven focus group interviews. Clinician specialties included 4 obstetricians, 8 anesthesiologists, 3 pediatricians, 14 OB nurses, and 5 OB technicians. Obstetric simulation groups from which study participants were recruited roughly approximated the clinician mix during an actual emergency. The mean clinician age was 41 years. Table 1 includes other subject demographics. The sample included clinicians at different experience levels within their specialty and with a range of years working at the hospital. All subjects had experience providing care in each of the hospital’s three ORs (OR-A, OR-B, OR-C).

**Table 1:** Demographic information by specialty.

Specialty	n	Gender ^a^	Age ^b^	Years of Experience ^c^	Years at LPCH ^c^
Anesthesiologist	8	62.5	33 (28–38)	3 (0–14)	1.5 (1–3)
Obstetrician	4	25.0	36.25 (28–51)	4.5 (0–24)	2 (1–20)
Pediatrician	3	100.0	50 (45–60)	19 (16–25)	19 (14–28)
OB Nurse	14	0.0	41.69 (25–64)	15 (3–40)	10 (2–31)
OB Technician	5	20.0	44.60 (29–58)	17 (3–29)	8 (1–29)

^a^ Values presented at percent male within each specialty.

^b^ Values presented as mean (range).

^c^ Values presented as median (range).

### Characteristics of the labor and delivery unit studied

**Fig 1:**
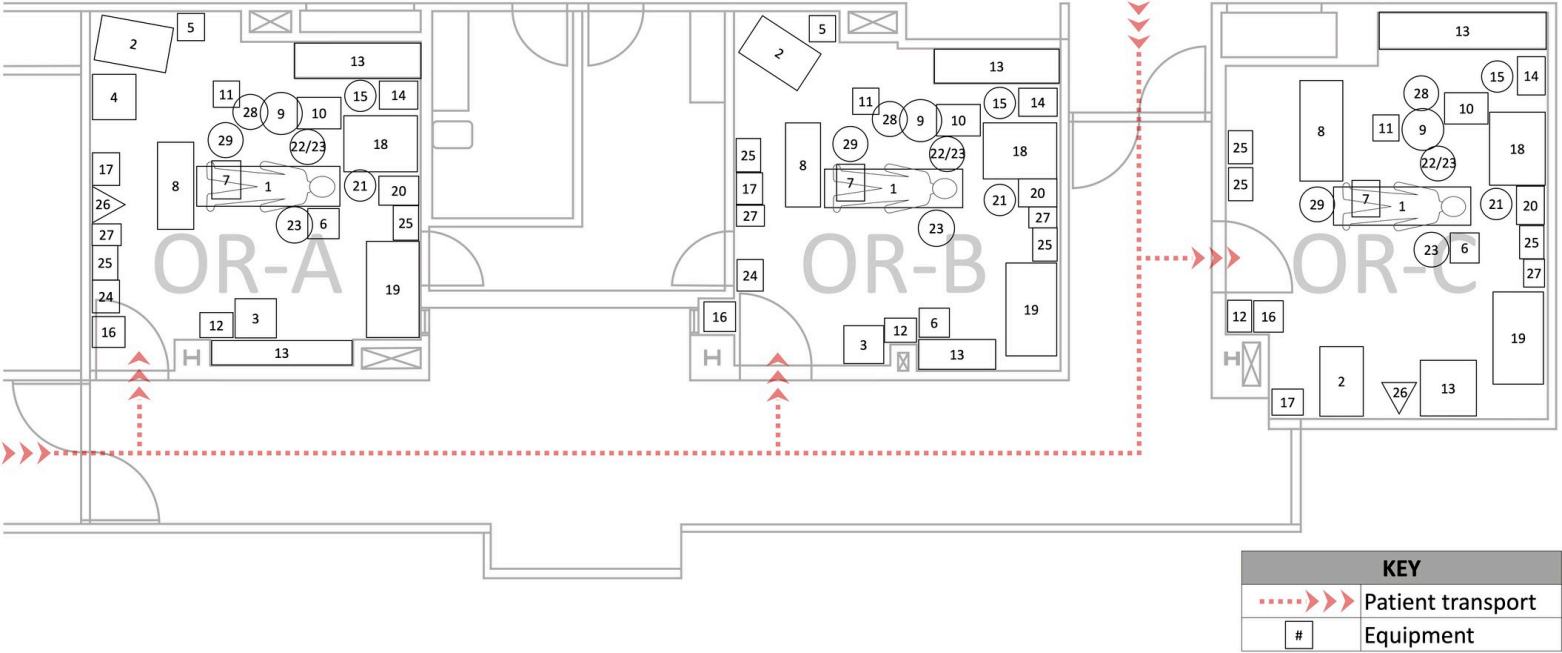
Layout and orientation of obstetric ORs and equipment. 1-OR table; 2-infant warmer; 3-oak cabinet or cart with fetal heart monitor; 4-neonatal code cart; 5-neonatal supplies cart; 6-support person chair; 7-mayo stand; 8-surgical back table; 9-suction apparatus; 10-electrocautery; 11-round bucket basin stand; 12-surgical steps; 13-built-in supply cabinet; 14-nurses’ computer workstation; 15-round rolling sitting stool; 16-hovermachine device; 17-prep stand for Foley; 18-anesthesia machine; 19-automated medication dispensing system; 20-anesthesia computer workstation; 21-anesthesiologist chair; 22-forced air warmer; 23-IV pole; 24-non-biohazard bin; 25-biohazard bin; 26-soiled linen bin; 27-sharps bin; 28-IV pole for surgical sponges; 29-kick bucket.

The institution studied has approximately 4,000–5,000 annual deliveries, with a Nulliparous, Term, Singleton, Vertex (NTSV) Cesarean rate of 21.9% [33]. The labor and delivery unit consists of 10 labor rooms and three ORs (OR-A, OR-B, OR-C) in a closed unit that is part of a tertiary care pediatrics hospital connected to an adult hospital. Each OR contains standard equipment for emergent and non-emergent Cesarean deliveries as shown in Fig 1. Fig 1 depicts the layout and orientation of the obstetric OR unit and scaled representations of equipment. At the institution studied, staff required during an emergency Cesarean includes the inhouse obstetric, anesthesiology, nursing, and neonatology teams. When an emergency Cesarean is called by the obstetrician there is notification by both group page and phone calls.

### Task analysis

**Fig 2:**
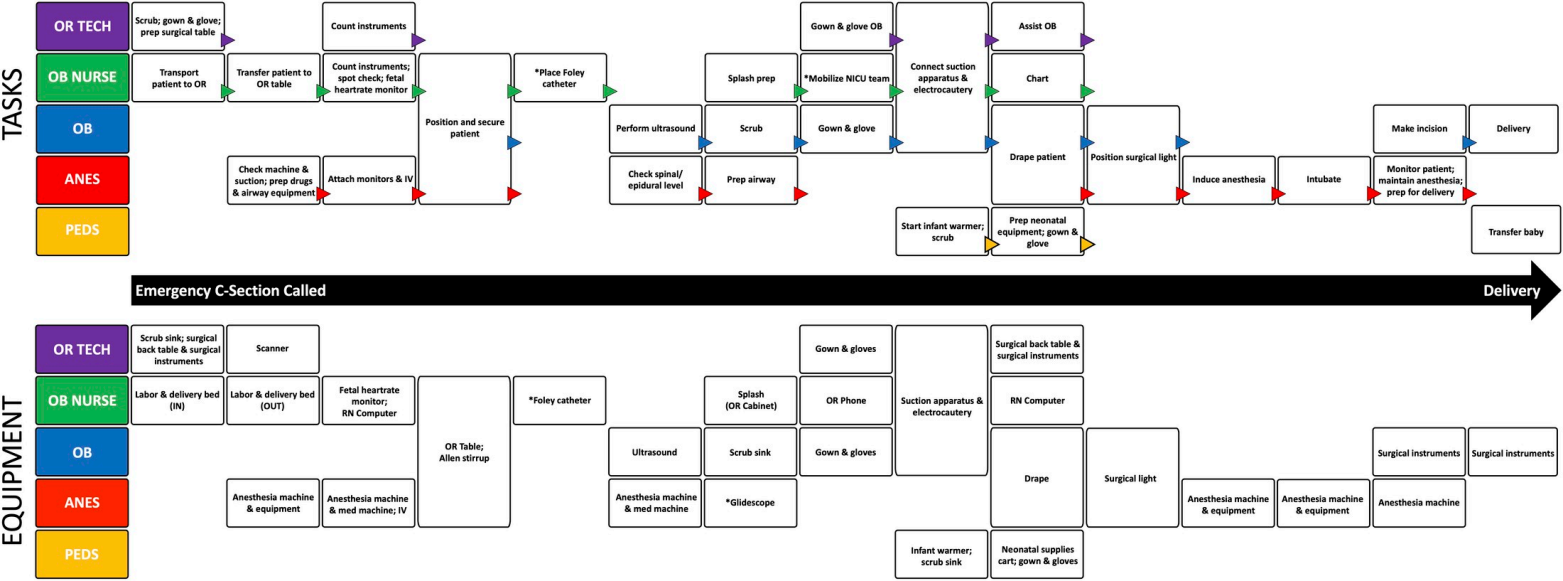
Task and equipment timeline for an emergency Cesarean.

A task timeline for an emergency Cesarean delivery and the equipment required is shown in Fig 2. It begins with the decision to perform an emergency Cesarean and ends with delivery of the fetus. The task analysis revealed complex interdependencies between teams, with 30 individual subspecialty and multidisciplinary task groupings and the use of more than 20 pieces of equipment. Tasks varied in complexity and were grouped based on how specialties perceived their dependency on other tasks. The number of grouped tasks per specialty ranged from 2 to 12. There were four tasks identified that required individuals from multiple specialties; 1) position and secure the patient, 2) set up suction and electrocautery, 3) drape the patient, and 4) position surgical lights. There were three key tasks or stopping points identified where all subsequent tasks to be performed by all specialties were reliant on those key tasks being completed: 1) position and secure the patient, 2) drape the patient, and 3) induction and intubation of the patient.

### Perceived areas of congestion and time spent by specialty

**Fig 3:**
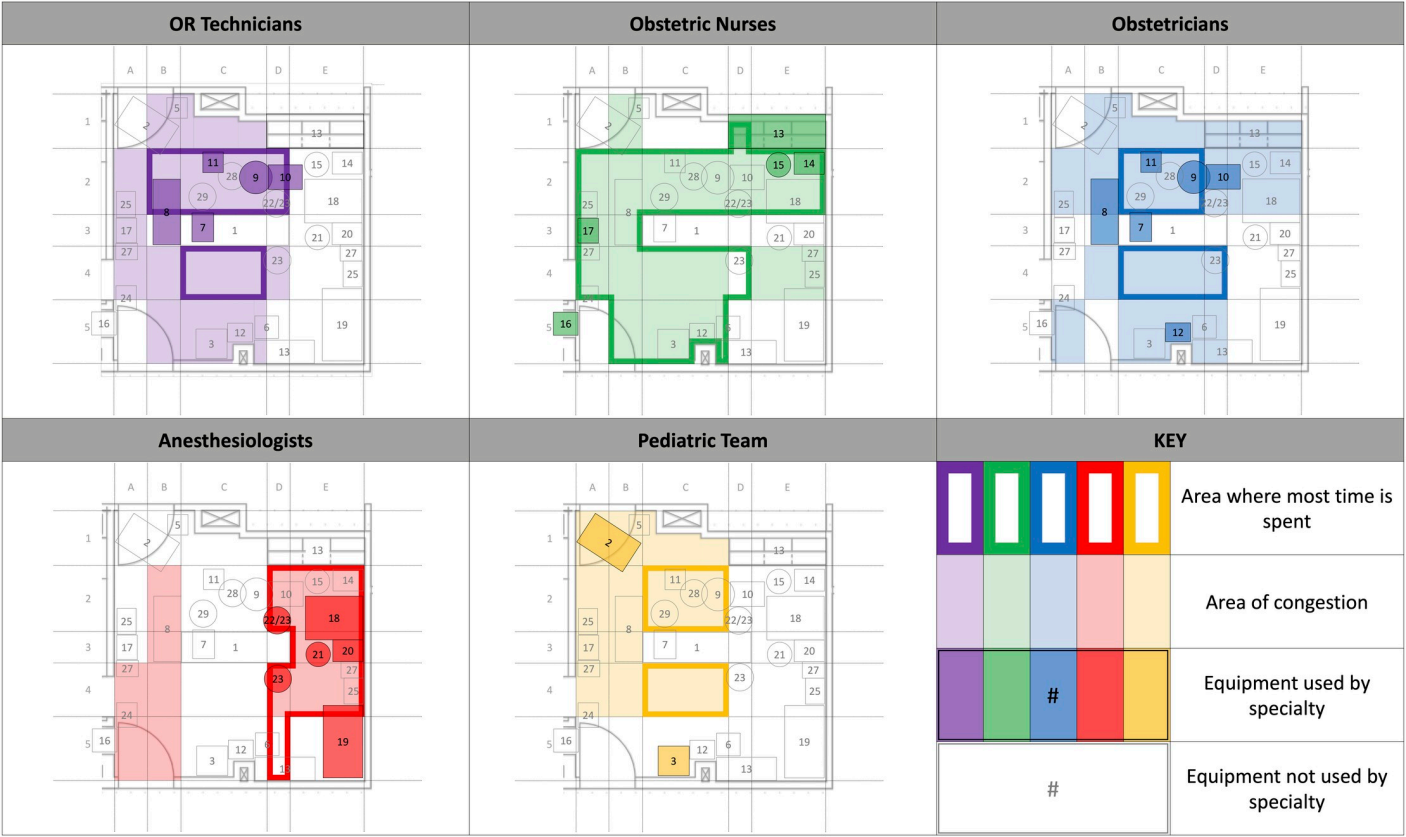
Reported areas of congestion and time spent in OR-B by specialty.

Fig 3 illustrates areas in OR-B where each discipline reported spending time and the areas that were congested during an emergency Cesarean delivery.

### Overlay of perceived areas of congestion and task location

**Fig 4:**
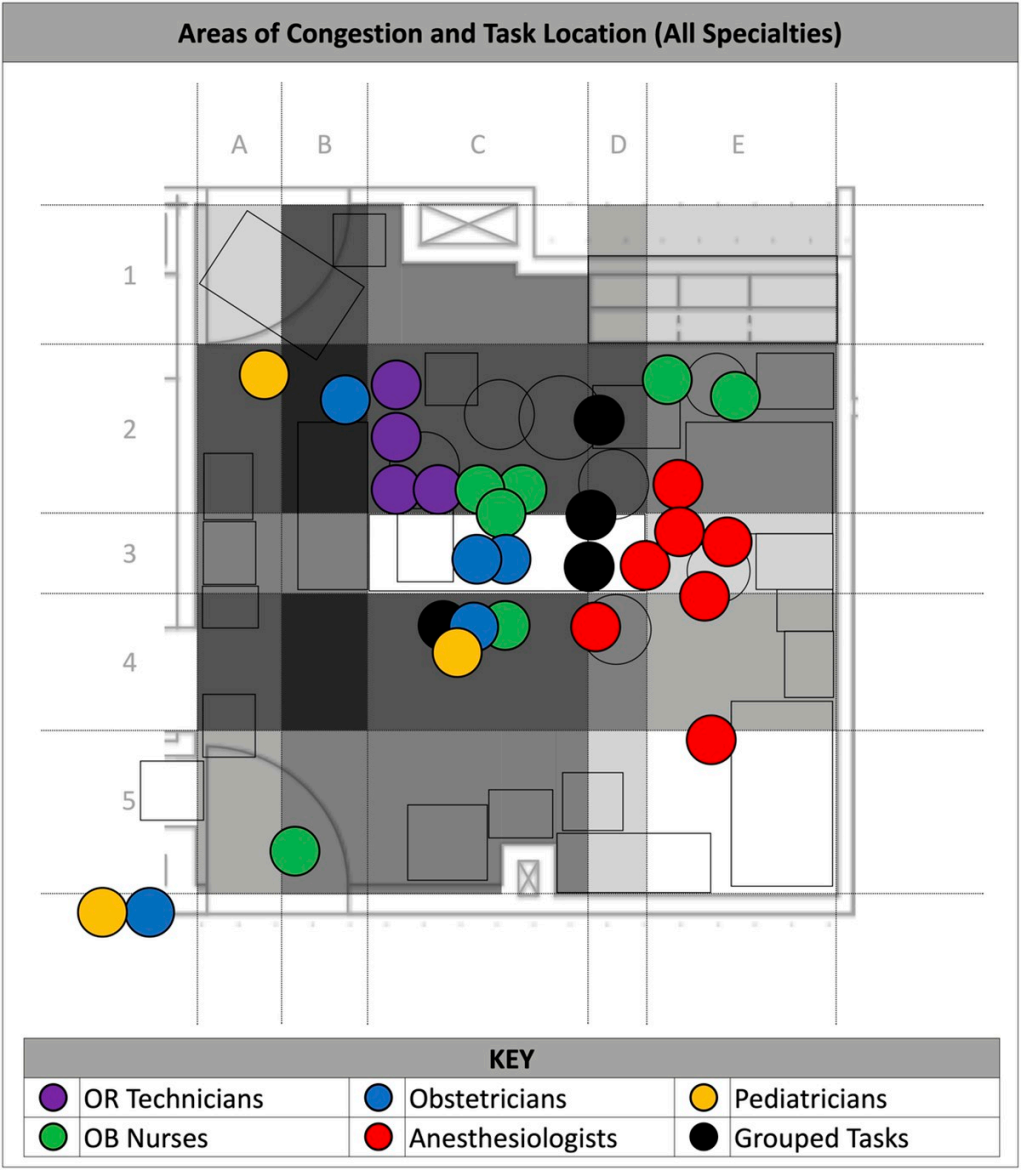
Overlay of reported areas of congestion and task location in OR-B. Non-shaded areas indicate no reports of congestion. Increasing degrees of shading correlate with increasing reports of congestion by one or more specialties. Colored dots indicate task groupings color coded by specialty.

The congestion heat map (Fig 4) illustrates combined areas of congestion across all specialties and approximate location of tasks.

### Qualitative analysis

**Fig 5:**
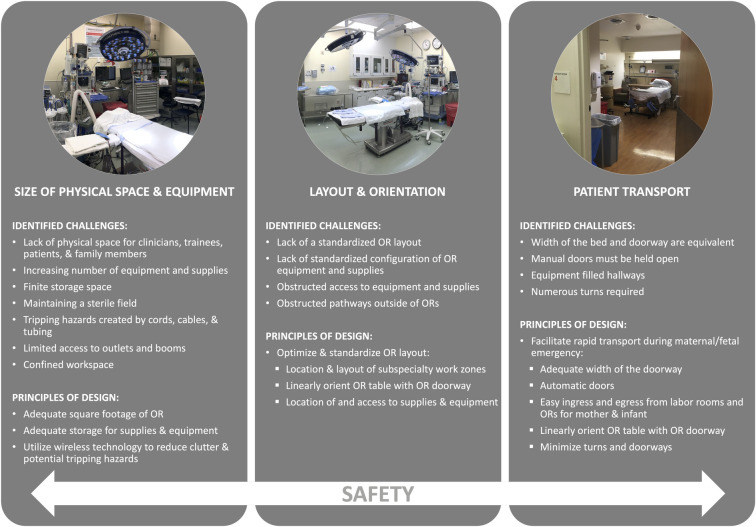
Key points: Identified challenges during an emergency Cesarean and relative principles of design.

Focus group data were distilled into three categories related to physical space of the OR and labor and delivery unit: 1) size of physical space, equipment, and clutter 2) layout and orientation, and 3) patient transport (Fig 5).

## Discussion

Emergency Cesarean is a complicated event that requires timely coordination of a multidisciplinary team to achieve efficient delivery, facilitate maternal and/or infant resuscitation, and optimize outcomes. We utilized validated techniques in the field of human factors to perform a participatory user-centered analysis of space utilization in the obstetric OR. Through this novel approach, we gained insights about the interdependence between specialties and identified design factors related to the physical space that may impact safety during an emergency Cesarean delivery.

To our knowledge, this is the first time that task and equipment analysis has been applied to emergency Cesarean delivery, revealing a dynamic interplay of tasks and equipment across teams. Tasks that include multiple sub-steps and multiple pieces of equipment, and involve multidisciplinary personnel underscore the complexity of the procedure. Availability, design, and location of equipment may impact task completion. Any delay in one task can have downstream effects, slowing delivery and potentially worsening outcomes for mother and/or newborn.

Heatmaps showed areas around the patient, especially to the patient’s left, right, and feet, were the most frequently reported areas of congestion across all specialties, which is not surprising given the goals of an emergency Cesarean delivery. Interestingly, every specialty indicated congestion in the areas where they spent most of their time. This suggests a human bias toward awareness of one’s own space over that of others. Overcoming this bias represents an opportunity for outside observers to study interactive workflow, define more efficient work zones, and reduce congestion while balancing the inherently high costs of square footage in the OR.

Qualitative observations from interviews added nuance to and expanded on our understanding of space utilization challenges specifically related to size of physical space and equipment, layout and orientation, and patient transport. Notably, optimal OR size has not yet been defined in the literature [14]. This presents a potential area for future research. While unplanned Cesarean deliveries are performed infrequently, rates of planned Cesarean deliveries in the U.S. and parts of the world remain high [34, 35]. The challenges highlighted in the interviews collected in this study suggest that space for additional personnel and equipment needed for an emergency Cesarean delivery should be considered in design guidelines for any obstetric OR. Designing for this more complex scenario may also improve the safety and efficiency of the more routine, planned Cesarean delivery.

From an ergonomics perspective, it is inherently inefficient that all three ORs in the institution studied are oriented in such a way that a labor bed that is being driven feet first needs to be backed into the OR head first to align with the OR table. During an emergency, it would be safer and more efficient to be able to wheel the labor bed into the OR feet first. Additionally, the congestion heat map shows the right side of the OR table is more congested, so having to transfer the patient from the left side of the OR table may be ideal.

There are several limitations to this work. Because this study reflects the experience of users at a single institution and standardization in hospital design is lacking, our findings may not be generalizable to other labor and delivery units. Also, since emergency Cesarean deliveries are a rare subset of deliveries, it is likely that our study subjects may have different levels of experience or recall bias. Nurses represented a larger proportion of subjects in the sample, so their opinions may be disproportionately represented. Furthermore, the perspectives of additional stakeholders such as patients, family members, hospital leadership, medical device manufacturers, medical architects, supply chain management, and custodial workers were not captured. The small number of healthcare professionals in this study also made it underpowered to detect quantitative subspecialty differences in perceptions regarding the impact of OR size, layout, and equipment availability on the performance of emergency Cesarean sections.

Future research on human factors and ergonomics methods should be conducted to better understand and improve the physical design of hospitals for all users and streamline complicated systems within healthcare. The interdependency between users of the space must be considered, because improving the efficiency of one group may not ultimately improve the efficiency of the entire procedure. For instance, improving the efficiency of surgical preparation by scrub technicians and obstetricians may not make a difference unless similar or better improvements are made for the anesthesiologist who is tasked with putting the patient under general anesthesia before surgical incision can take place.

Overall, our findings support existing evidence that the labor and delivery unit environment varies widely across the U.S. and that differences in design may impact care [7, 8, 36, 37]. Testing different configurations of ORs and even entire labor and delivery units during actual and simulated obstetric emergencies would allow researchers to measure how design may impact performance and care delivery. Multicenter analyses including all stakeholders while considering differences in practice and resource levels could further define the optimal design principles for maximizing safety and efficiency in labor and delivery units.

## Supporting information

S1 Table Frequencies (percentages) of responses on impact of equipment availability, OR orientation and size on facilitating emergency Cesareans by specialty. (DOCX)

S2 Table Kruskal-Wallis test to assess for differences in responses on impact of equipment availability, OR orientation, and OR size in facilitating an emergency Cesarean by specialty. (DOCX)

S1 Appendix Survey instrument. (DOCX)

S2 Appendix Semi-structured interview instrument. (DOCX)

S3 Appendix Stanford University IRB research information sheet. (DOCX)

S4 Appendix Provider demographic information questionnaire. (DOCX)

S1 File Collated survey responses. (XLSX)
