# Supplementary material for: Single-center task analysis and user-centered assessment of physical space impacts on emergency Cesarean delivery
Source: PLoS One. 2021 Jun 10;16(6):e0252888. doi: 10.1371/journal.pone.0252888 (PMC8191948; doi:10.1371/journal.pone.0252888)
Supplement: S1 Table — (DOCX) [file pone.0252888.s001.docx]

**S1 Table. Frequencies (percentages) of responses on impact of equipment availability, OR orientation and size on facilitating emergency Cesareans by specialty.**

| **Equipment Availability** ^a^ | | | | | | | | | | | |
| --- | --- | --- | --- | --- | --- | --- | --- | --- | --- | --- | --- |
| **Specialty** | **n** | **Agree** | | | **Neutral** | | | **Disagree** | | | |
| Anesthesiologists  Obstetricians  Pediatricians  OB Nurses  OB Technicians | 8 (23.5)  4 (11.8)  3 (8.8)  14 (41.2)  5 (14.7) | 6 (75.0)  3 (75.0)  0 (0.0)  11 (78.6)  4 (80.0) | | | 2 (25.0)  1 (25.0)  1 (33.3)  2 (14.3)  0 (0.0) | | | 0 (0.0)  0 (0.0)  2 (66.7)  1 (7.1)  1 (20.0) | | | |
| **TOTAL** | 34 (100.0) | 24 (70.6) | | | 6 (17.6) | | | 4 (11.8) | | | |
| **Orientation** ^a^ | | | | | | | | | | | |
| **Specialty** | **n** | **OR-A**^b^ | | | **OR-B** | | | **OR-C** | | | |
|  |  | **Agree** | **Neutral** | **Disagree** | **Agree** | **Neutral** | **Disagree** | **Agree** | **Neutral** | **Disagree** | |
| Anesthesiologists  Obstetricians  Pediatricians  OB Nurses  OB Technicians | 8 (23.5)  4 (11.8)  3 (8.8)  14 (41.2)  5 (14.7) | 1 (14.3)  2 (50.0)  0 (0.0)  1 (7.1)  0 (0.0) | 3 (42.9)  1 (25.0)  0 (0.0)  2 (14.3)  2 (40.0) | 3 (42.9)  1 (25.0)  3 (100.0)  11 (78.6)  3 (60.0) | 1 (12.5)  0 (0.0)  0 (0.0)  7 (50.0)  1 (20.0) | 4 (50.0)  4 (100.0)  0 (0.0)  3 (21.4)  1 (20.0) | 3 (37.5)  0 (0.0)  3 (100.0)  4 (28.6)  3 (60.0) | 6 (75.0)  4 (100.0)  1 (33.3)  12 (85.7)  3 (60.0) | 2 (25.0)  0 (0.0)  0 (0.0)  1 (7.1)  1 (20.0) | 0 (0.0)  0 (0.0)  2 (66.7)  1(7.1)  1 (20.0) | |
|  | 34 (100.0) | 4 (12.1) | 8 (24.2) | 21 (63.6) | 9 (26.5) | 12 (35.3) | 13 (38.2) | 26 (76.5) | 4 (11.8) | 4 (11.8) | |
| **Size** ^a^ | | | | | | | | | | | |
| **Specialty** | **n** | **OR-A** | | | **OR-B** | | | **OR-C** | | | |
|  |  | **Agree** | **Neutral** | **Disagree** | **Agree** | **Neutral** | **Disagree** | **Agree** | **Neutral** | | **Disagree** |
| Anesthesiologists  Obstetricians  Pediatricians  OB Nurses  OB Technicians | 8 (23.5)  4 (11.8)  3 (8.8)  14 (41.2)  5 (14.7) | 0 (0.0)  1 (25.0)  0 (0.0)  1 (7.1)  1 (20.0) | 3 (37.5)  2 (50.0)  0 (0.0)  0 (0.0)  1 (20.0) | 5 (62.5)  1 (25.0)  3 (100.0)  13 (92.9)  3 (60.0) | 1 (12.5)  1 (25.)  0 (0.0)  3 (21.4)  2 (40.0) | 4 (50.0)  3 (75.0)  0 (0.0)  5 (35.7)  0 (0.0) | 3 (37.5)  0 (0.0)  3 (100.0)  6 (42.9)  3 (60.0) | 8 (100.0)  3 (75.0)  1 (33.3)  12 (85.7)  4 (80.0) | 0 (0.0)  1 (25.0)  0 (0.0)  1 (7.1)  0 (0.0) | | 0 (0.0)  0 (0.0)  2 (66.7)  1 (7.1)  1 (20.0) |
| **TOTAL** | 34 (100.0) | 3 (8.8) | 6 (17.6) | 25 (73.5) | 7 (20.6) | 12 (35.3) | 15 (44.1) | 28 (82.4) | 2 (5.9) | | 4 (11.8) |

^a^ Values presented as frequency (percentage)

^b^ Percentages are calculated using n = 33 (Anesthesiologists, n = 7)
