# Supplementary material for: Single-center task analysis and user-centered assessment of physical space impacts on emergency Cesarean delivery
Source: PLoS One. 2021 Jun 10;16(6):e0252888. doi: 10.1371/journal.pone.0252888 (PMC8191948; doi:10.1371/journal.pone.0252888)
Supplement: S2 Table — (DOCX) [file pone.0252888.s002.docx]

**S2 Table. Kruskal-Wallis test to assess for differences in responses on impact of equipment availability, OR orientation, and OR size in facilitating an emergency Cesarean by specialty.**

| **Parameter** | **Specialty** | **Mean Rank** | **df** | **χ^2^** | ***P*-value** |
| --- | --- | --- | --- | --- | --- |
| **Equipment Availability** | Anesthesiologists  Obstetricians  Pediatricians  OB Nurses  OB Technicians | 16.25  16.25  30.83  16.70  16.50 | 4 | 9.203 | 0.056 |
| **OR-A Orientation** | Anesthesiologists^a^  Obstetricians  Pediatricians  OB Nurses  OB Technicians | 13.86  9.13  23.00  19.46  17.20 | 4 | 7.510 | 0.111 |
| **OR-B Orientation** | Anesthesiologists  Obstetricians  Pediatricians  OB Nurses  OB Technicians | 18.88  15.50  28.00  13.82  20.90 | 4 | 6.961 | 0.138 |
| **OR-C Orientation** | Anesthesiologists  Obstetricians  Pediatricians  OB Nurses  OB Technicians | 17.25  13.50  26.17  15.93  20.30 | 4 | 6.666 | 0.155 |
| **OR-A Size** | Anesthesiologists  Obstetricians  Pediatricians  OB Nurses  OB Technicians | 16.19  9.25  22.00  20.57  14.90 | 4 | 8.662 | 0.070 |
| **OR-B Size** | Anesthesiologists  Obstetricians  Pediatricians  OB Nurses  OB Technicians | 17.38  11.13  27.00  17.25  17.80 | 4 | 5.085 | 0.279 |
| **OR-C Size** | Anesthesiologists  Obstetricians  Pediatricians  OB Nurses  OB Technicians | 14.50  18.25  26.50  16.86  18.10 | 4 | 7.444 | 0.114 |

^a^ One anesthesiologist did not respond to this survey item.
