# Supplementary material for: Single-center task analysis and user-centered assessment of physical space impacts on emergency Cesarean delivery
Source: PLoS One. 2021 Jun 10;16(6):e0252888. doi: 10.1371/journal.pone.0252888 (PMC8191948; doi:10.1371/journal.pone.0252888)
Supplement: S1 Appendix — (DOCX) [file pone.0252888.s003.docx]

Thank you for agreeing to participate.

Please help us by filling out this questionnaire.

Please answer these questions focusing on **Stat C-Sections** only.

Feel free to skip any questions you don’t feel you can answer or add additional comments in the margins.

| **Background Questions:** |
| --- |
| 1. Which of the three operating rooms at LPCH would you prefer to use in the event of a **STAT C-Section**? |
| ☐ OR LDR-A (#2746/2839)  ☐ OR LDR-B (#2742/2831)  ☐ OR LDR-C (#2735/2827) |
| 1. What do you like about working in that OR for a Stat C-Section? |
|  |

| **Physical Design and Layout** | | |
| --- | --- | --- |
| 1. The orientation of the OR relative to the door facilitates the speed of performing a STAT C-Section: | | |
| **OR-A** | **OR-B** | **OR-C** |
| ☐ Strongly Disagree  ☐ Disagree  ☐ Neutral  ☐ Agree  ☐ Strongly Agree | ☐ Strongly Disagree  ☐ Disagree  ☐ Neutral  ☐ Agree  ☐ Strongly Agree | ☐ Strongly Disagree  ☐ Disagree  ☐ Neutral  ☐ Agree  ☐ Strongly Agree |
| 1. The size of the OR facilitates the speed of performing a STAT C-Section: | | |
| **OR-A** | **OR-B** | **OR-C** |
| ☐ Strongly Disagree  ☐ Disagree  ☐ Neutral  ☐ Agree  ☐ Strongly Agree | ☐ Strongly Disagree  ☐ Disagree  ☐ Neutral  ☐ Agree  ☐ Strongly Agree | ☐ Strongly Disagree  ☐ Disagree  ☐ Neutral  ☐ Agree  ☐ Strongly Agree |
| 1. The medical equipment availability in the OR facilitates a STAT C-Section: | | |
| ☐ Strongly Disagree  ☐ Disagree  ☐ Neutral  ☐ Agree  ☐ Strongly Agree | | |

| **Space Background** *We’re going to focus on OR-B for a moment.* |
| --- |
| 1. During a STAT C-Section where do you spend most of your time? *Mark locations in the green squares on the map of OR-B below with an Asterix * Feel free to add arrows for movement.* |
| \|  \| \| --- \|  1. During a STAT C-Section what are some places that get congested for you in OR-B? *Mark locations on the map of OR-B below with a #.* |
| 1. From a space perspective, what are the biggest problems with a Stat C-Section? |


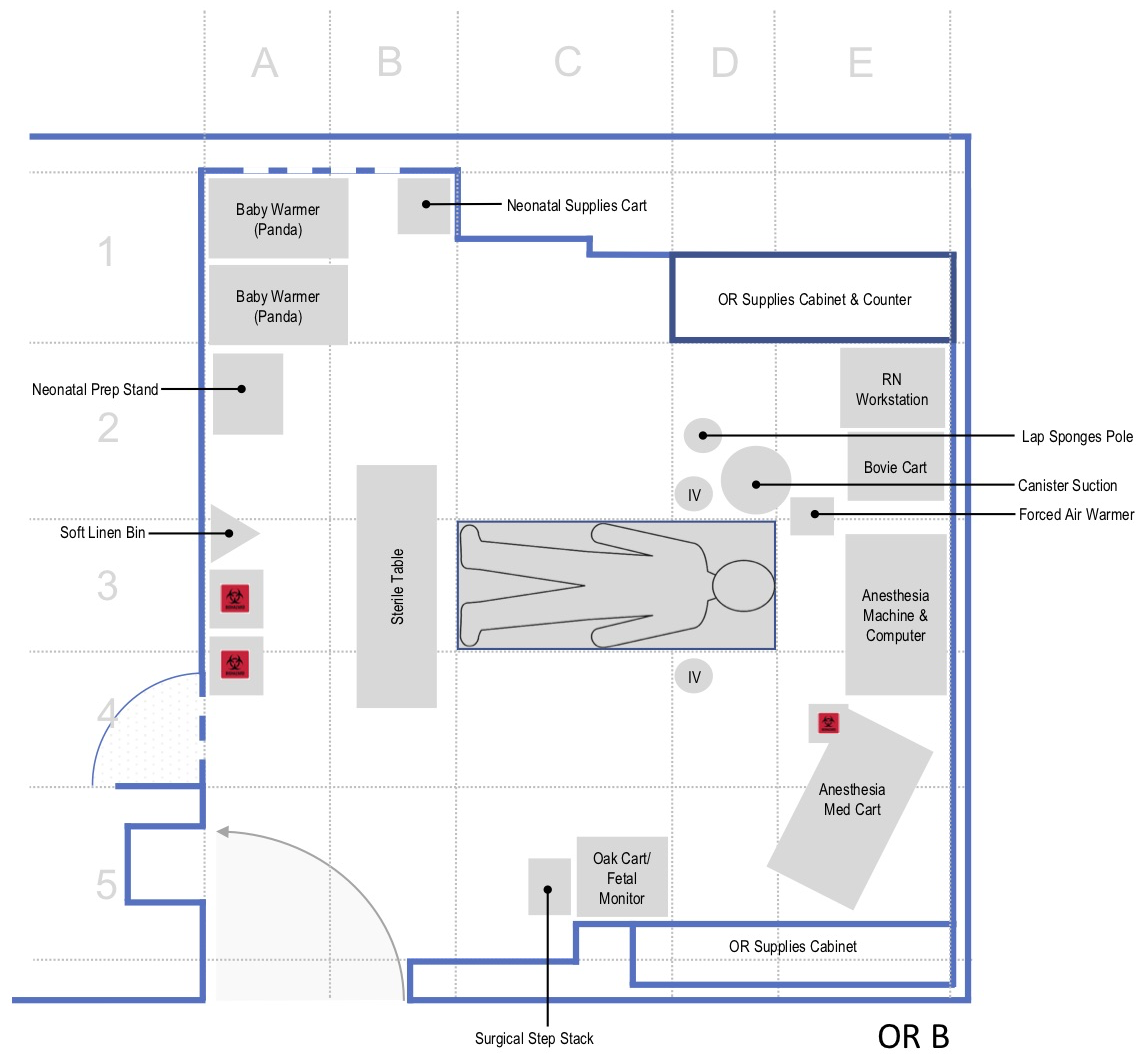


|  |  |  |  |  |  |  |  |  |  |
| --- | --- | --- | --- | --- | --- | --- | --- | --- | --- |
|  |  |  |  |  |  |  |  |  |  |
|  |  |  |  |  |  |  |  |  |  |
|  |  |  |  |  |  |  |  |  |  |
|  |  |  |  |  |  |  |  |  |  |
|  |  |  |  |  |  |  |  |  |  |
|  |  |  |  |  |  |  |  |  |  |
|  |  |  |  |  |  |  |  |  |  |
|  |  |  |  |  |  |  |  |  |  |
|  |  |  |  |  |  |  |  |  |  |

| **Demographic Information** *This just helps us understand who we’ve met.* |
| --- |
| 1. What is your specialty at Lucile Packard (LPCH)? |
| ☐ Anesthesiologist  ☐ Obstetrician/Gynecologist  ☐ Pediatrician  ☐ Nurse  ☐ Technician  ☐ Other (please describe) |
| 1. Within your specialty what is your position |
| ☐ Attending  ☐ Fellow  ☐ Resident  ☐ Intern  ☐ Tech  ☐ Other (please describe) |
| 1. Which operating room(s) at LPCH have you worked in (check all that apply)? |
| ☐ OR LDR-A (#2746/2839)  ☐ OR LDR-B (#2742/2831)  ☐ OR LDR-C (#2735/2827) |
| 1. Not counting school, how many years of experience do you have in this specialty? |
|  |
| 1. How many years have you worked at LPCH? |
|  |
| 1. What is your usual shift? |
| ☐ Days  ☐ Evenings  ☐ Variable Shifts |
| 1. What is your age? |
|  |
| 1. What is your gender? |
| ☐ Male  ☐ Female  ☐ Prefer not to answer |
| 1. Any additional comments? |
|  |
| 1. Have we interviewed you previously? |
| ☐ Yes  ☐ No |

Thank you for your time!
