# Supplementary material for: Single-center task analysis and user-centered assessment of physical space impacts on emergency Cesarean delivery
Source: PLoS One. 2021 Jun 10;16(6):e0252888. doi: 10.1371/journal.pone.0252888 (PMC8191948; doi:10.1371/journal.pone.0252888)
Supplement: S2 Appendix — (DOCX) [file pone.0252888.s004.docx]

| **Visit 1 Interview Guide** |
| --- |
| **Opening Script:** |
| As a reminder, my name is [name]. Just to set expectations, I will be asking you some background questions, then I would like to understand your job so we can possibly make it easier.  I will be focusing on STAT C-Sections specifically, and I want to understand the equipment you use, where in the OR you spend most of your time, and a little about the tasks.  I will ask you to walk us through your tasks and equipment you use to perform a STAT C-Section.  *Equipment used by different teams, when and why.*  *Space used by different teams, when and why.*  *Dependencies among tasks.*  I want to remind you that:   - There are no right or wrong answers; - You can decline to answer any question or ask us to stop at any time during the interview; - And that the information you provide will not compromise your job at Lucile Packard Children’s Hospital.   May we proceed? Now I would like to start with some background questions. |

| **Background Questions:** |
| --- |
| 1. What are your positions at Lucile Packard (LPCH)?   ☐ Anesthesiologist  ☐ Attending ☐ Fellow ☐ Resident ☐ Intern ☐ Tech  ☐ Obstetrician/Gynecologist  ☐ Attending ☐ Fellow ☐ Resident ☐ Intern ☐ Tech  ☐ Pediatrician  ☐ Attending ☐ Fellow ☐ Resident ☐ Intern ☐ Tech  ☐ Nurse  ☐ Technician  ☐ Other: |

| **Space Background**  *We’re going to focus on OR-B for a moment.* |
| --- |
| 1. From a space perspective, what are the biggest problems with a STAT C-Section? |

| **Physical Design and Layout** |
| --- |
| 1. From a space perspective, what parts of the transportation to the OR cause the most problems and why? *Where do you bump into things?* |
| 1. How could you fix these things? |


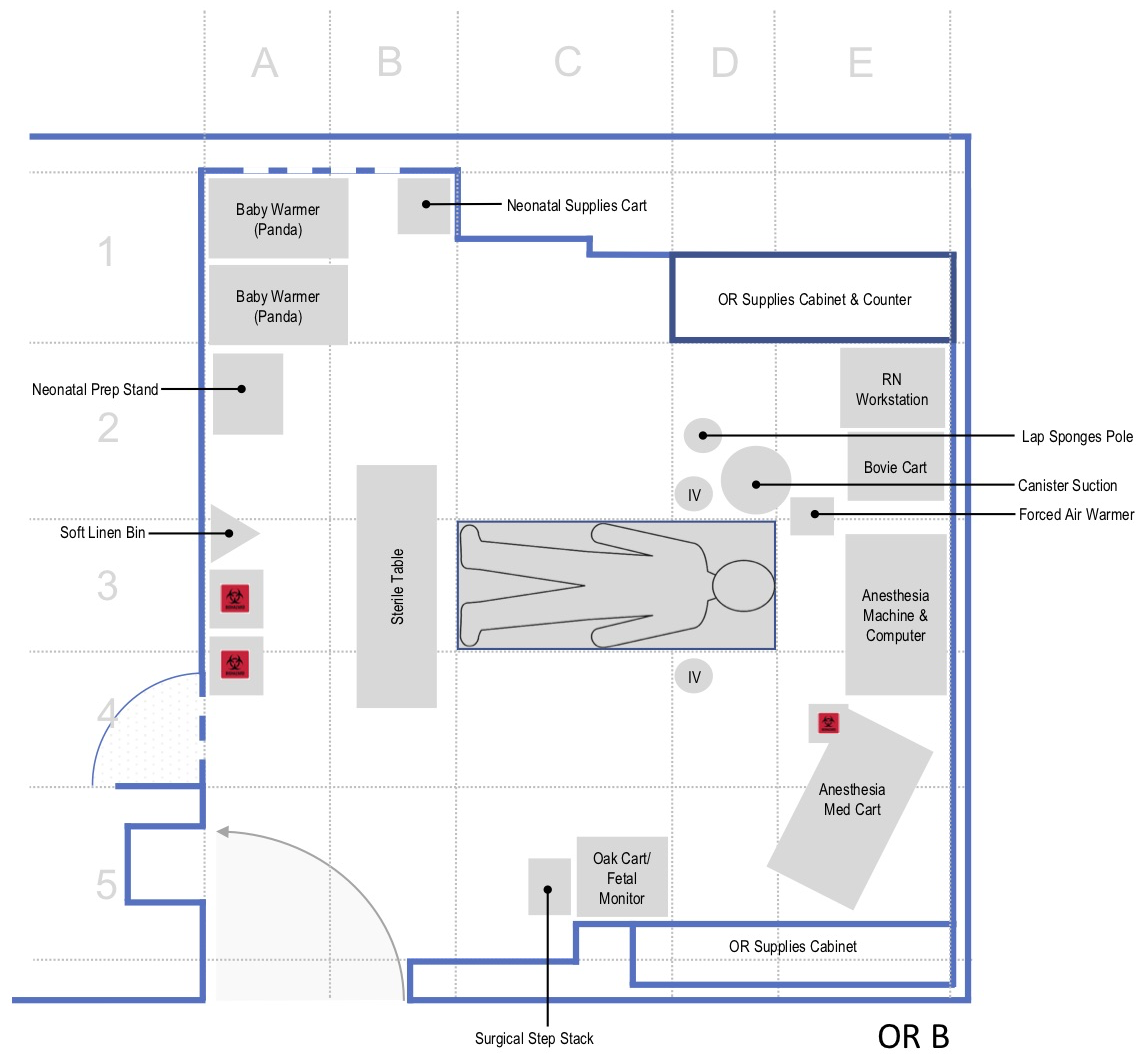


| 1. Walk me through the tasks you perform from the decision to move to a STAT C-Section through delivery. *(Use blueprint of OR as a visual guide; List high-level group tasks in Task Inventory Sheet)* |
| --- |
| 1. Without being too detailed, what equipment is used for this task? |
| 1. Does the equipment move for this task? Where to where? |
| 1. Who moves the equipment? |
| 1. What tasks by other people need to be done before you can do this? |
| 1. What tasks are waiting for you to finish this task? |

| **Physical Design and Layout** |
| --- |
| 1. How does the size of the OR impact your workflow? |

| **Closing Script** |
| --- |
| Before concluding this interview, is there anything you would like to add?  Thank you for spending this time and sharing your information with us. If you have any questions after we leave, you can contact Naola or Lillian |
