# Supplementary material for: Single-center task analysis and user-centered assessment of physical space impacts on emergency Cesarean delivery
Source: PLoS One. 2021 Jun 10;16(6):e0252888. doi: 10.1371/journal.pone.0252888 (PMC8191948; doi:10.1371/journal.pone.0252888)
Supplement: S3 Appendix — (DOCX) [file pone.0252888.s005.docx]

**DESCRIPTION:** You are invited to participate in **a research study** on how the physical design of the labor and delivery suite may affect patient and worker safety.

You will be asked to either 1) let us shadow you as you provide care to patients, 2) answer a few questions regarding your view on the physical design elements of the labor and delivery suite, 3) provide a tour of the facilities in the labor and delivery suite, or 4) all of the above activities.

The National Institutes of Health (NIH) is providing financial support and/or material for this study.

**TIME INVOLVEMENT:** Your participation will take approximately 60-120 minutes for the observation/shadowing, 60-120 minutes for the tour, and 5-10 minutes for the survey.

**RISKS AND BENEFITS:** The risks associated with this study are minimal. There are no benefits associated with this study but your participation may help to improve patient safety for mothers and babies during labor and delivery. We cannot and do not guarantee or promise that you will receive any benefits from this study. Your decision whether or not to participate in this study will not affect your employment.

**PAYMENTS:** You will not receive monetary compensation for your participation.

**SUBJECT'S RIGHTS:** If you have read this form and have decided to participate in this project, please understand your **participation is voluntary** and you have the **right to withdraw your consent or discontinue participation at any time without penalty or loss of benefits to which you are otherwise entitled**. **The alternative is not to participate.** You have the right to refuse to answer particular questions. Your individual privacy will be maintained in all published and written data resulting from the study.

This research is covered by a Certificate of Confidentiality from the National Institutes of Health. The researchers with this Certificate may not disclose or use information, documents, or biospecimens that may identify you in any federal, state, or local civil, criminal, administrative, legislative, or other action, suit, or proceeding, or be used as evidence, for example, if there is a court subpoena, unless you have consented for this use. Information, documents, or biospecimens protected by this Certificate cannot be disclosed to anyone else who is not connected with the research except, if there is a federal, state, or local law that requires disclosure (such as to report child abuse or communicable diseases but not for federal, state, or local civil, criminal, administrative, legislative, or other proceedings, see below); if you have consented to the disclosure, including for your medical treatment; or if it is used for other scientific research, as allowed by federal regulations protecting research subjects.

The Certificate cannot be used to refuse a request for information from personnel of the United States federal or state government agency sponsoring the project that is needed for auditing or program evaluation by the Agency for Health Research and Quality (AHRQ), which is funding this project or for information that must be disclosed in order to meet the requirements of the federal Food and Drug Administration (FDA). You should understand that a Certificate of Confidentiality does not prevent you from voluntarily releasing information about yourself or your involvement in this research. If you want your research information released to an insurer, medical care provider, or any other person not connected with the research, you must provide consent to allow the researchers to release it.

**CONTACT INFORMATION:**

*Questions:* If you have any questions, concerns or complaints about this research, its procedures, risks and benefits, contact the Protocol Director, Dr. Naola Austin at (206) 257-8459 or naola@stanford.edu.

*Independent Contact:* If you are not satisfied with how this study is being conducted, or if you have any concerns, complaints, or general questions about the research or your rights as a participant, please contact the Stanford Institutional Review Board (IRB) to speak to someone independent of the research team at (650)-723-5244. You can also write to the Stanford IRB, Stanford University, 3000 El Camino Real, Five Palo Alto Square, 4th Floor, Palo Alto, CA 94306.

This Research Information Sheet is for you to keep.

**If you agree to participate in this research, please indicate this to the researcher.**
