# Supplementary material for: Single-center task analysis and user-centered assessment of physical space impacts on emergency Cesarean delivery
Source: PLoS One. 2021 Jun 10;16(6):e0252888. doi: 10.1371/journal.pone.0252888 (PMC8191948; doi:10.1371/journal.pone.0252888)
Supplement: S4 Appendix — (DOCX) [file pone.0252888.s006.docx]

## Provider Demographic Information Questionnaire

**Name**:___________________________________________________________

**Date:** ________________

**1. Job Title: Please specify your credentials.**

## □ DO □ MD □ MS

## □ PA □ RN □ Surg. Tech □ Other________

**2. If applicable, please indicate your training status:**

⬜ Resident (year) ____ ⬜ Attending

⬜ Fellowship (year) ____ ⬜ Other (please specify)________

**3. What is your primary specialty?**

## □ Pediatrics □ Obstetrics □ Midwifery

## □ Anesthesiology □ Other: ______________________

**4. How many cumulative years of experience do you have in this**

**specialty? ________**

***** BELOW QUESTIONS ARE OPTIONAL*****

## 5. Age or Please specify the year of your birth: __________

## 6. Sex: Please specify your sex. ⬜ Female ⬜ Male

## ⬜ Other (please specify)________________

**7. Ethnicity origin: Please specify your ethnicity.**

## ⬜ Hispanic/Latino ⬜ Non-Hispanic ⬜ Prefer not to answer

## 8. Race: Please specify your race.

## ⬜ Asian ⬜ Black or African American

## ⬜ Native American or American Indian ⬜ White

⬜ Hispanic/Latino ⬜ More than one race

⬜ Other ⬜ Prefer not to answer

**9. Education: Please specify your highest education level.**

## □ High school □ Bachelor’s degree

## □ Master’s degree □ Doctoral degree

**For internal use**

⬜ Updated Excel Participant ID:

⬜ Updated OnCore Subject/OnCore ID:

Focus Group Code:
